# Comprehensive evaluation of taste dysfunction in allogeneic hematopoietic cell transplant recipients: a combined subjective and objective assessment

**DOI:** 10.1007/s00520-026-10403-9

**Published:** 2026-02-13

**Authors:** Yoko Tsukamoto, Saori Oku, Junichi Yamazoe, Yoshiko Imamura, Tsugiyo Nakamura, Haruna Hikita, Mikiko Nakamura, Emi Taniyama, Takuji Yamauchi, Yasuo Mori, Haruhiko Kashiwazaki

**Affiliations:** 1 Dental Hygiene Section, Department of Medical Technology, Kyushu University Hospital, Kyushu University, Fukuoka, Japan; 2 Section of Geriatric Dentistry and Perioperative Medicine in Dentistry, Division of Maxillofacial Diagnostic and Surgical Sciences, Faculty of Dental Science, Kyushu University, 3-1-1 Maidashi, Higashi-Ku, Fukuoka, 812-8582 Japan; 3 Department of Nursing, Kyushu University Hospital, Kyushu University, Fukuoka, Japan; 4 Department of Medicine and Biosystemic Science, Kyushu University Graduate School of Medical Sciences, Fukuoka, Japan

**Keywords:** Taste dysfunction, Allogeneic hematopoietic cell transplantation, Whole-mouth taste testing method, Chemotherapy-induced Taste Alterations Scale, Umami

## Abstract

**Purpose:**

Taste dysfunction is a common but underrecognized complication in patients undergoing allogeneic hematopoietic cell transplantation (allo-HCT). Taste dysfunction can adversely affect oral intake, nutritional status, and overall quality of life. This study aimed to comprehensively evaluate taste function in allo-HCT recipients using both objective and subjective measures and to identify clinical factors associated with taste disturbances.

**Methods:**

We conducted a prospective observational study of 21 adult allo-HCT recipients. Taste function was assessed at two time points (pre-conditioning and pre-discharge) using the whole-mouth taste testing method for the five basic tastes and the Chemotherapy-induced Taste Alteration Scale (CiTAS). All 21 patients completed the CiTAS, and 19 patients underwent whole-mouth taste testing.

**Results:**

Most patients exhibited objective taste dysfunction before transplantation, particularly for sweet, salty, sour, and bitter taste qualities. The objective taste thresholds remained stable post-transplantation, but the subjective CiTAS scores worsened for basic taste, general alterations, and discomfort. There was a trend (*p* = 0.057) for objective umami taste to be better preserved in patients with gastrointestinal graft-versus-host disease (GI-GVHD). Oral mucositis was associated with higher phantogeusia/parageusia scores, while high malnutrition risk or weight loss correlated with lower subjective symptom scores.

**Conclusion:**

The discrepancy between subjective and objective assessments of taste dysfunction highlights that taste perception is regulated by multifactorial and complex mechanisms. Intensive supportive care, which is often provided to severely ill patients, may offer psychological reassurance that indirectly improves self-reported taste symptoms. Whether umami sensitivity is influenced by systemic factors such as GI-GVHD requires further study.

**Supplementary Information:**

The online version contains supplementary material available at 10.1007/s00520-026-10403-9.

## Introduction

Allogeneic hematopoietic cell transplantation (allo-HCT) is a potentially curative treatment for various hematologic malignancies and inherited hematopoietic disorders [1]. Conditioning regimens, comprising high-dose chemotherapy and/or total body irradiation, aim to eradicate residual malignant cells and immune effector cells that may cause graft rejection. These treatments often lead to mucosal injury and myelosuppression, which contribute to various oral complications, including taste dysfunction [2]. Taste disorders are particularly impactful, as they reduce quality of life and can contribute to appetite loss, decreased oral intake, and weight loss [3]. The resultant malnutrition is associated with poorer transplant outcomes, such as increased risks of acute graft-versus-host disease (GVHD) and non-relapse mortality, and worse progression-free and overall survival rates [4].

Taste alterations occur in 56–76% of patients undergoing cancer treatment [5]. A recent review reported that the peak rates of objective and subjective taste dysfunction in HCT recipients were 21.4% and 20–100%, respectively [6]. Although zinc supplementation is effective in some patients with zinc deficiency [7], there is no standardized prophylactic or therapeutic strategy for taste dysfunction in patients with cancer [5]. Subjective and objective taste dysfunction are most common during the neutropenic phase (7–14 days after initiation of conditioning), and taste function usually recovers within 2–3months after engraftment, suggesting a direct impact of the conditioning regimen [6]. Umami and salt tastes tend to be most affected in allo-HCT recipients, with bitter taste least affected [6]. Factors thought to influence taste changes in allo-HCT recipients include oral mucositis [8], hyposalivation [8], alterations in the oral and/or gut microbiota due to broad-spectrum antibiotic and antifungal use [9], tissue damage from GVHD [10], and melphalan-based conditioning regimens [11]. Taste disorders may persist beyond the turnover period of taste bud cells [12], suggesting a distinct pathophysiology from that observed after standard chemotherapy. Although several studies have evaluated taste dysfunction in allo-HCT recipients [13–22], the assessment methods have varied and often been inconsistent. For example, none of those ten studies utilized both objective and subjective assessments, those employing objective tests failed to evaluate all five basic taste qualities [13–17], and the subjective tests varied widely between studies and were generalized quality-of-life/adverse effect instruments with little focus on taste disturbance [18–22]. Conventional tools may not sufficiently capture the broad spectrum of taste dysfunction symptoms, including hypogeusia, hypergeusia, and dysgeusia [16, 23].

This study aimed to comprehensively assess taste dysfunction in allo-HCT recipients by combining an objective evaluation via the whole-mouth taste testing method with a subjective assessment using the Chemotherapy-induced Taste Alteration Scale (CiTAS) [24]. We also analyzed the clinical factors associated with taste dysfunction in this population. The combined use of validated objective and subjective assessments provided novel insights into taste dysfunction in allo-HCT recipients, supporting the use of this integrated approach in the evaluation of taste disorders.

## Methods

### Study design and participants

We prospectively enrolled adult patients (aged ≥ 18 years-old) who underwent allo-HCT at Kyushu University Hospital between March 1, 2023 and November 12, 2024. The exclusion criteria were death or transferal to another hospital before discharge.

Clinical data extracted from the electronic medical records included age, sex, underlying disease, conditioning regimen, GVHD prophylaxis, body mass index, graft source, number of days to neutrophil engraftment, number of remaining teeth, presence/absence of oral mucositis, denture use, oral intake status, weight change during the previous 3–6 months, and presence of GVHD or gastrointestinal (GI)-GVHD. The presence/absence of oral mucositis was evaluated at least once weekly, from the beginning of the conditioning regimen until engraftment, by two dental professionals using the National Cancer Institute Common Toxicity Criteria (NCI-CTCAE) version 3.0. Nutritional status was assessed using the Malnutrition Universal Screening Tool (MUST) [25], which includes three components: body mass index (0 points: > 20 kg/m^2^; 1 point: 18.5–20 kg/m^2^; 2 points: < 18.5 kg/m^2^), weight loss in the past 3–6 months (0 points: < 5%; 1 point: 5–10%; 2 points: > 10%), and acute disease effect (2 points if no oral nutritional intake was expected for > 5 days).

Taste assessments were performed at two time points: before conditioning (mean ± standard deviation [SD]: 11.0 ± 3.0 days pre-transplantation) and prior to discharge (50.5 ± 15.9 days post-transplantation). Subjective (CiTAS) and objective (whole-mouth taste test) assessments were conducted on the same day at each time point to ensure comparability between the two measures. These two time points were selected because whole-mouth taste testing requires repeated rinsing with taste solutions, which is feasible only when the patient is able to attend a clinic (so the evaluation can be performed in a standardized, controlled setting) and their nausea has settled. At our institution, discharge is permitted when oral intake and daily activities have improved sufficiently to allow the patient to live independently at home; therefore, the pre-discharge taste assessment was conducted at that point. Oral care was routinely performed before conditioning and immediately prior to discharge, which aligned with the timing of the taste assessments.

### Transplant procedures

All patients received conditioning regimens and GVHD prophylaxis. Oral levofloxacin (500 mg/day) was initiated from the beginning of the conditioning protocol to prevent bacterial infection. Oral fluconazole or intravenous echinocandins and oral acyclovir were administered as prophylaxis against fungal infections and herpes simplex virus reactivation, respectively. All patients received professional oral care using mechanical cleaning devices in the dental treatment room prior to conditioning therapy. Bedside oral assessments and hygiene care were provided daily from the day of transplantation until neutrophil engraftment.

Standard nutritional care was provided under the supervision of a multidisciplinary nutrition support team (NST). When oral intake was possible, the patient was provided with regular hospital meals, excluding grapefruit and products containing live lactic acid bacteria (e.g., Yakult, Mirumiru, and yogurt). The patient was also encouraged to eat suitable items they purchased themselves from mobile food outlets. Oral nutritional supplements were added if appetite decreased. Enteral or parenteral nutrition was initiated if oral intake became difficult due to transplant-related adverse events (e.g., generalized fatigue or oral mucositis). Additionally, at the request of a physician or nurse, a registered dietitian provided individualized nutritional intervention when a patient encountered difficulties with oral food/nutrient intake. Multidisciplinary information sharing was conducted during NST meetings on the ward. The target energy intake was 25–30 kcal/kg/day with a protein intake of 1.2–1.5 g/kg/day, in accordance with established guidelines [26]. The patient was discharged when they were able to take their oral medications and food independently, showed no signs of acute GVHD, and had recovered sufficiently to resume their normal daily activities at home.

### Objective taste test: whole-mouth taste testing

The assessment was performed as described previously [27, 28]. Five basic taste qualities (sweet, salty, sour, bitter, and umami) were evaluated using three concentrations (low, medium, and high) of the following solutions: sucrose (1.56, 12.5, and 100 mM), NaCl (1.56, 12.5, and 100 mM), hydrochloric acid (0.156, 1.25, and 10 mM), quinine-hydrochloride (1.56, 12.5, and 100 μM), and monosodium glutamate (1.56, 12.5, and 100 mM). Each solution (2 mL) was administered via a pipette, and the participant was instructed to swish it around the mouth, spit it out, and identify the perceived taste using a standardized evaluation form (Supplementary Table 1). The perception threshold for each taste quality was defined as the lowest concentration at which the correct taste was identified on two consecutive trials. Taste sensitivity was categorized as hypersensitive (recognition at the low concentration), normal (recognition at the medium concentration), or hyposensitive (recognition only at the high concentration or failure to recognize the taste). In this study, both hypersensitivity and hyposensitivity were considered indicative of taste dysfunction.

### Subjective taste test: CiTAS

Subjective taste function was assessed using the CiTAS, a validated instrument developed to evaluate chemotherapy-induced taste disorders [24]. The CiTAS comprises 18 questions grouped into four categories: decline in basic taste, general taste alterations, phantogeusia and parageusia, and discomfort (Supplementary Table 2). Each item is rated on a 5-point Likert scale (1 to 5), and the mean score for each category is calculated. Higher mean scores indicate a greater degree of taste dysfunction within the respective category.

### Statistical analysis

Normally distributed data are described as mean ± SD. Non-normally distributed variables are described as median (range). McNemar’s test was used to compare the incidence of taste disorder before and after transplantation. Changes in CiTAS scores across the four categories were analyzed with the Wilcoxon signed-rank test. The possible associations of oral mucositis, GI-GVHD, malnutrition risk, and weight loss with post-transplant CiTAS scores were analyzed using the Mann–Whitney U test, Kruskal–Wallis test, and one-way analysis of variance (ANOVA). Fisher’s exact test was utilized to examine the associations between various clinical factors (overall survival, oral mucositis, fasting status, melphalan, GI-GVHD, use of steroids, malnutrition risk and weight loss) with umami taste disorder. All statistical analyses were performed using SPSS version 28.0 (IBM Japan, Tokyo). Statistical significance was defined as a *p*-value < 0.05.

### Ethics statement

All participants were fully informed about the nature and purpose of the study and provided written informed consent prior to enrollment. This study was approved by the Ethics Committee of Kyushu University Hospital (approval number: 22262) and conducted in accordance with the Declaration of Helsinki.

## Results

### Participant characteristics

Twenty-seven adult patients who underwent allo-HCT during the study inclusion period were initially enrolled. After excluding five patients who died during the study period and one patient who was transferred to another hospital, 21 patients (57.5 ± 12.5 years-old; 15 males, 76.2%) were included in the final analysis. The patient characteristics are summarized in Table 1. Taste assessments were performed 11.0 ± 3.0 days before transplantation and 50.5 ± 15.9 days after transplantation (prior to discharge). All 21 patients completed the CiTAS, while 19 patients underwent whole-mouth taste testing (two patients were not assessed due to severe nausea and a deterioration in their condition). The median (range) time from transplantation to discharge from hospital was 52 (36–127) days and did not differ significantly between patients with GI-GVHD and those without GI-GVHD (55.5 [46.0–127.0] days vs 52.0 [36.0–115.0] days, *p* = 0.334).

**Table 1:** Characteristics of the study participants (*n* = 21)

Characteristic		Value
Age (years), mean ± SD		57.5 ± 12.5
Gender, *n* (%)	Male	15 (71.4)
	Female	6 (28.6)
Underlying disease, *n* (%)	Acute myeloid leukemia	9 (42.9)
	Acute lymphoblastic leukemia	3 (14.3)
	Malignant lymphoma	2 (9.5)
	Myelodysplastic syndrome	2 (9.5)
	Others	5 (23.8)
Melphalan dose in conditioning regimen, *n* (%)	None	9 (42.9)
	80 mg/m^2^	7 (33.3)
	140 mg/m^2^	5 (23.8)
Graft-versus-host disease prophylaxis, *n* (%)	CI + sMTX	5 (23.8)
	CI + MMF ± PTCy	13 (61.9)
	Others	3 (14.3)
Total body irradiation before transplantation, *n* (%)	Yes	21 (100)
	No	0 (0)
Graft source, *n* (%)	Bone marrow	2 (9.5)
	Peripheral blood stem cell	16 (76.2)
	Cord blood	3 (14.3)
Disease status prior to transplantation, *n* (%)	CR1/CR2/CR3	8 (38.1)/1 (4.8)/1 (4.8)
	SD/non-CR	1 (4.8)/5 (23.8)
	NC	5 (23.8)
Prior history of irradiation	Yes	2 (9.5)
	No	19 (90.5)
Neutrophil engraftment (days), mean ± SD		17.0 ± 2.7
Number of teeth, mean ± SD		25.0 ± 5.1
Oral mucositis, *n* (%)	Yes	8 (38.1)
	No	13 (61.9)
Denture use, *n* (%)	Yes	17 (81.0)
	No	4 (19.0)
Fasting status, *n* (%)	Yes	8 (38.1)
	No	13 (61.9)
Gastrointestinal graft-versus-host disease, *n* (%)	Yes	6 (28.6)
	No	15 (71.4)
Use of steroids, *n* (%)	Yes	8 (38.1)
	No	13 (61.9)
Malnutrition risk, *n* (%)	Low	3 (14.3)
	Medium	6 (28.6)
	High	12 (57.1)
Time from transplant to discharge (days), median (range)		52 (36–127)

*CI*, calcineurin inhibitor; *CR*, complete remission; *MMF*, mycophenolate mofetil; *NC*, not classified (cases involving immune disorders rather than hematologic malignancies); *PTCy*, post-transplant cyclophosphamide; *SD*, stable disease; *sMTX*, short-term methotrexate

### Incidence of objective taste dysfunction

There was a high prevalence of taste dysfunction for sweet, salty, sour, and bitter taste qualities at baseline. Sweet taste dysfunction was observed in 89.5% (17/19) of patients before transplantation (hyposensitivity in 84.2% and hypersensitivity in 5.3% of patients). Salty, sour and bitter taste disorders were observed in 94.7% (18/19), 94.7% (18/19) and 89.5% (17/19) of patients, respectively, before transplantation (hyposensitivity in all cases). Interestingly, the post-transplantation prevalences of sweet taste disorder (94.7%, 18/19), salty taste hyposensitivity (84.2%, 16/19), sour taste hyposensitivity (94.7%, 18/19), and bitter taste hyposensitivity (89.5%, 17/19) were not significantly different to the pre-transplantation values.

**Fig. 1:**
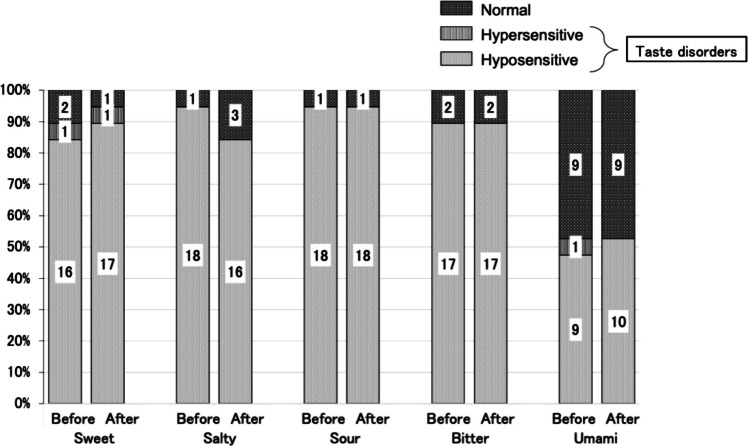
Results of the objective taste assessments (whole-mouth taste testing method). For all five taste qualities, the incidence of taste dysfunction after allogeneic hematopoietic cell transplantation (After) was not significantly different to that before allogeneic hematopoietic cell transplantation (Before). McNemar’s test was used for the statistical analyses

By contrast, umami taste was better preserved at both the pre-transplantation and post-transplantation time points. Umami taste disorder was present in 52.6% (10/19) of patients both before and after transplantation. One patient exhibited hypersensitivity and nine showed hyposensitivity before transplantation, while all 10 cases of umami taste dysfunction after transplantation were classified as hyposensitivity (Fig. 1). Notably, there was not complete overlap between the groups of patients with umami taste disorders before and after transplantation; some showed improvement after transplantation, while others exhibited newly developed umami taste dysfunction.

### Incidence of subjective taste dysfunction

**Fig. 2:**
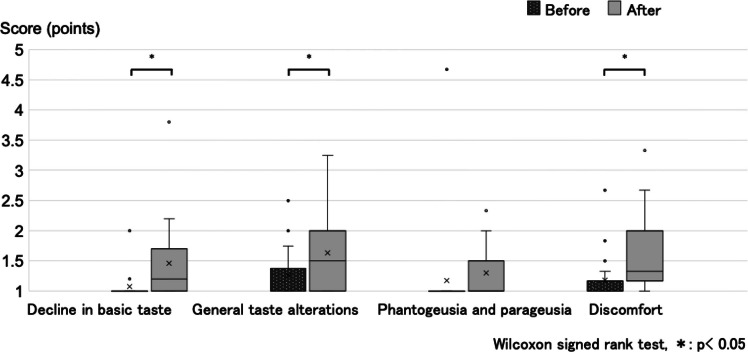
Results of the subjective taste assessments (Chemotherapy-induced Taste Alteration Scale). The box plots show the median, interquartile range, range, mean, and outliers. * *p* < 0.05, after allogeneic hematopoietic cell transplantation (After) vs before allogeneic hematopoietic cell transplantation (Before)

Median scores for three of the four CiTAS subscales increased significantly following transplantation: decline in basic taste (median [range]: from 1.00 [1.00–2.00] before transplantation to 1.20 [1.00–3.80] after transplantation, *p* = 0.007), general taste alterations (1.00 [1.00–2.50] to 1.50 [1.00–3.25], *p* = 0.025), and discomfort (1.00 [1.00–2.67] to 1.33 [1.00–3.33], *p* = 0.003; Fig. 2). There was no significant change in the phantogeusia and parageusia subscale score (1.00 [1.00–4.67] to 1.00 [1.00–2.33], *p* = 0.072). These findings indicate that subjective taste disturbances worsened following allo-HCT.

### Associations between clinical factors and objective/subjective taste dysfunction

Since there was incomplete overlap between the groups of patients with umami taste disorders before and after transplantation, we explored whether objective umami taste dysfunction after transplantation was associated with various clinical factors (Table 2). No significant associations were observed between objective umami taste dysfunction and overall survival, oral mucositis, steroid use, melphalan administration, fasting status, malnutrition risk, recent weight loss or GI-GVHD (Table 2), although there was a trend toward patients who developed GI-GVHD being more likely to retain normal umami taste perception after transplantation than those without GI-GVHD (*p* = 0.057).

**Table 2:** Univariable analysis of clinical factors associated with objective umami taste dysfunction

Variable		Preserved umami taste (*n* = 9)	Impaired umami taste (*n* = 10)
Survival outcome	Alive (*n* = 15)	7	8
	Dead (*n* = 4)	2	2
	*p*-value	1.000	
Oral mucositis	Yes (*n* = 8)	4	4
	No (*n* = 11)	5	6
	*p*-value	1.000	
Fasting status	Yes (*n* = 7)	4	3
	No (*n* = 12)	5	7
	*p*-value	0.650	
Gastrointestinal graft-versus-host disease	Yes (*n* = 6)	5	1
	No (*n* = 13)	4	9
	*p*-value	0.057	
Use of steroids	Yes (*n* = 8)	3	5
	No (*n* = 11)	6	5
	*p*-value	0.650	
Melphalan	Yes (*n* = 8)	7	3
	No (*n* = 11)	2	7
	*p*-value	0.071	
Malnutrition risk	Low (*n* = 3)/Medium (*n* = 6)	5	4
	High (*n* = 10)	4	6
	*p*-value	0.179	
Recent weight loss	< 5% (*n* = 4)	3	1
	≥ 5% (*n* = 15)	6	9
	*p*-value	0.303	

Analysis performed using Fisher’s exact test

Next, we examined the relationship between CiTAS scores at discharge (subjective taste dysfunction) and potential systemic and local factors (Table 3). Patients with oral mucositis had significantly higher scores for the phantogeusia and parageusia subscale than those without mucositis (1.50 [1.00–2.33] vs 1.00 [1.00–2.00], *p* = 0.008). Discomfort subscale scores were significantly lower in patients with GI-GVHD than in those without (1.00 [1.00–1.50] vs 1.66 [1.17–3.33], *p* = 0.006). Participants at high or medium risk for malnutrition exhibited significantly lower scores for the phantogeusia and parageusia subscale than those at low risk (1.00 [1.00–1.67] and 1.00 [1.00–2.33] vs 2.00 [1.67–2.00], *p* = 0.042). Patients with < 5% weight loss in the past 3 months had higher scores for the decline in basic taste subscale than those with 5–10% or ≥ 10% weight loss (2.10 [1.20–3.80] vs 1.00 [1.00–1.60] and 1.10 [1.00–2.20], *p* = 0.036). Additionally, there was a trend toward the general taste alterations score being higher for participants with < 5% weight loss compared to those with 5–10% or ≥ 10% weight loss (2.25 [1.75–3.25] vs 1.50 [1.00–2.25] and 1.25 [1.00–2.50], *p* = 0.054). Therefore, patients at high risk of malnutrition or with greater weight loss tended to report fewer/milder subjective taste-related complaints (Table 3), even though nutritional risk and recent weight loss did not correlate with objective umami thresholds (Table 2).

**Table 3:** Univariable analysis of clinical factors associated with subjective taste dysfunction

Variable		Decline in basic taste	General taste alterations	Phantogeusia and parageusia	Discomfort
	Yes (*n* = 8)	1.10 (1.00–2.20)	1.75 (1.00–2.50)	1.50 (1.00–2.33)	1.58 (1.00–3.33)
	No (*n* = 13)	1.20 (1.0–3.80)	1.25 (1.00–3.25)	1.00 (1.00–2.00)	1.33 (1.00–2.50)
	*p-*value	0.547	0.301	0.008	0.238
Gastrointestinal graft-versus-host disease ^a^	Yes (*n* = 6)	1.20 (1.00–2.20)	1.75 (1.00–2.50)	1.00 (1.00–1.67)	1.00 (1.00–1.50)
	No (*n* = 15)	1.20 (1.00–3.80)	1.50 (1.00–3.25)	1.33 (1.00–2.33)	1.66 (1.17–3.33)
	*p-*value	0.970	0.791	0.622	0.006
Malnutrition risk ^b^	Low (*n* = 3)	2.00 (1.20–2.20)	2.00 (1.75–2.50)	2.00 (1.67–2.00)	1.33 (1.17–1.50)
	Medium (*n* = 6)	1.10 (1.00–1.20)	1.37 (1.00–2.25)	1.00 (1.00–2.33)	1.25 (1.00–3.33)
	High (*n* = 12)	1.10 (1.00–3.80)	1.37 (1.00–3.25)	1.00 (1.00–1.67)	1.66 (1.00–2.67)
	*p-*value	0.159	0.238	0.042	0.601
Recent weight loss	< 5% (*n* = 4)				
	5–10% (*n* = 9)				
	> 10% (*n* = 8)				
	*p-*value	0.036	0.054	0.205	0.152

Data are presented as median (range). ^a^ Mann-Whitney U test, ^b^ Kruskal-Wallis test, ^c^ one-way ANOVA

## Discussion

This prospective study comprehensively evaluated taste function in allo-HCT recipients using both objective and subjective assessments. Notably, the vast majority of patients exhibited impaired perception across four of the five basic taste qualities prior to transplantation, with umami taste being relatively preserved. By contrast, most participants exhibited no subjective perception of taste impairment. This seemingly contradictory finding reflects a dissociation between objective taste thresholds and subjective awareness. It was beyond the scope of this study to evaluate the reasons for this apparent discrepancy, but possibilities include: (1) the loss in gustatory taste dysfunction being masked by normal olfactory function (since smell contributes to taste perception); (2) a lack of awareness of objective taste abnormality due to gradual onset; and (3) unconscious compensation for taste loss by the patient (e.g., by adding extra herbs/spices). Our results suggest that objective and subjective assessments capture different dimensions of taste function and should be interpreted in a complementary manner. Post-transplantation evaluations revealed no marked deterioration in objective taste disorders but a worsening of subjective symptoms, particularly in basic taste perception, general alterations, and discomfort. This divergence suggests that subjective awareness of taste dysfunction may increase after transplantation despite minimal changes in measurable thresholds, underscoring the multifactorial and complex nature of taste perception in clinical settings.

The prevalence of taste disturbances before transplantation was markedly higher in our study than previously reported values of 11% to 31% [6]. The peak period for taste dysfunction is the early post-transplant neutropenic phase, which is often accompanied by severe mucositis. During this time, self-reported taste disorder rates range from 20% to over 50% [6], while objective assessments have identified dysgeusia in 21.4% and hypogeusia in 66.6% of patients [16]. Although taste function generally recovers within three months after transplantation [13], many of our patients continued to experience taste impairment at the time of discharge, which coincides with this recovery period. These discrepancies highlight the potential influence of differences in patient background, including prior treatment history and possible racial variations, in taste perception, warranting further investigation in future studies.

Salty taste, which is associated with electrolyte intake, is thought to be particularly susceptible to impairment both before and after transplantation [29]. An increased recognition threshold for salty taste after transplantation may make food taste “bland,” contributing to decreased appetite [30, 31]. Although we observed a higher incidence of taste disturbances than previous studies [6, 16], pre-transplant objective perception of umami (associated with protein intake) was normal in a substantial subset of our participants. Previous studies have indicated that glutamic acid, a key component of umami, promotes salivary secretion and protects the oral mucosa [32], and that umami receptors (T1R1/TIR3) tend to recover relatively early after chemotherapy [31, 33].

While glucocorticoids influence appetite regulation via hypothalamic orexigenic pathways and reduce inflammatory damage to peripheral tissues [34, 35], our study did not demonstrate an association between systemic corticosteroid use and objective umami taste preservation. The association between GI-GVHD and objective umami taste function was also not significant (*p* = 0.057), although it cannot be excluded that our study was underpowered to detect a real association between these factors. Future studies with larger sample sizes are needed to clarify whether umami perception is better preserved in patients with GI-GVHD, and if so, investigate the underlying mechanisms.

We also evaluated other potential factors influencing taste dysfunction. CiTAS scores for the phantogeusia and parageusia subscale were higher for patients at low risk of malnutrition (manageable with standard interventions) than for those at high risk (requiring intervention by nutrition support teams). These findings differ from those of a previous study using the Mini Nutritional Assessment Short-Form, which reported a higher incidence of malnutrition among patients with taste disorders [36]. The discrepancy may be attributed not only to differences in assessment tools but also to the possibility that patients in the high-risk group in our study received more comprehensive medical support, which may have reduced their self-reported discomfort. A similar trend was observed in relation to weight loss: patients with ≥ 5% weight loss tended to report lower scores for taste disturbances. This further supports the concept that patients receiving more intensive nutritional support may report fewer subjective complaints. Improved psychological well-being (e.g., reduced anxiety and stress), achieved through intensified support, may help mitigate against the subjective perception of taste-related symptoms [37–39].

Consistent with previous studies [40], patients with oral mucositis exhibited significantly higher CiTAS scores for phantogeusia and parageusia, as well as a tendency toward a worsening of the discomfort score post-transplantation. Oral mucositis causes pain, dysphagia, and reduced salivary secretion. These symptoms can impair oral intake, and previous studies have shown that fasting can increase the recognition threshold for sweet taste while decreasing that for salty taste [41]. Furthermore, xerostomia due to reduced salivary flow has been associated with the development of taste dysfunction [42, 43]. We have reported that cryotherapy during high-dose melphalan conditioning can mitigate against oral mucosal injury [44, 45], potentially reducing long-term taste impairment.

This study has several limitations. First, there are potential issues inherent to the methods used. Whole-mouth taste testing was difficult to implement in cases with nausea and fatigue. Furthermore, some CiTAS items relate to discomfort from food intake, making accurate assessment challenging in patients unable to ingest food orally. Second, the possible impact of pre-transplantation treatments must be considered. The prevalence of objective taste dysfunction before transplantation was higher than that reported previously, which introduces the risk of not fully capturing (i.e., underestimating) the changes in taste dysfunction attributable to transplantation. Future studies should consider controlling for patient background and possibly limiting the study population to cases where pre-transplantation taste dysfunctions are not prominent. Third, objective taste testing could not be performed during the neutropenic phase, when taste dysfunction is most pronounced, due to the presence of severe adverse events such as fatigue, nausea, and mucositis associated with the conditioning regimen. This limited our ability to evaluate temporal changes in taste dysfunction in detail. Fourth, the patient population was heterogeneous in terms of underlying diseases, conditioning regimens, and disease course, which may restrict the generalizability of the findings. Fifth, this was a single-center study with a small number of participants. However, it should be noted that previous investigations evaluating taste dysfunction after allo-HSCT have also generally had small sample sizes (around 20 patients) [13], highlighting the practical challenges of conducting clinical studies with large sample sizes in this field. Nevertheless, caution is required when generalizing the present results, and further validation in other centers is warranted. Future investigations could expand the sample size through multicenter collaborative research and by including patients undergoing autologous peripheral blood stem cell transplantation. Sixth, the follow-up period was short, so additional studies are needed to establish the longer-term effects of allo-HCT on objective and subjective taste dysfunction.

## Conclusion

Taste dysfunction is common in patients undergoing allo-HCT even before transplantation, and subjectively perceived taste disturbances tend to worsen after transplantation. Our study findings indicate that there may be complex and multifactorial influences on taste dysfunction in allo-HCT recipients and support the value of using both subjective and objective assessments alongside relevant clinical variables when evaluating taste disorders. Comprehensive supportive care, including nutritional and oral management, may help mitigate against subjective taste disturbances and improve overall patient quality of life.

Analysis performed using Fisher’s exact test.

## Supplementary Information

Below is the link to the electronic supplementary material.

ESM 1 (DOCX 28.4 KB)

## Data Availability

The data that support the findings of this study are available from the corresponding author upon reasonable request.
