# Supplementary material for: Comprehensive evaluation of taste dysfunction in allogeneic hematopoietic cell transplant recipients: a combined subjective and objective assessment
Source: Support Care Cancer. 2026 Feb 13;34(3):192. doi: 10.1007/s00520-026-10403-9 (PMC12901097; doi:10.1007/s00520-026-10403-9)
Supplement: Supplementary file 1 — (DOCX 28.4 KB) [file 520_2026_10403_MOESM1_ESM.docx]

**Supplementary Table 1 Taste quality evaluation sheet**

| What do you taste? |
| --- |
| 1. No taste at all |
| 2. No clearly identifiable taste |
| 3. Salty – like the ocean |
| 4. Sweet – like sugar |
| 5. Bitter – like coffee |
| 6. Sour – like lemon |
| 7. Umami – savory, like soup |

**Supplementary Table 2 Chemotherapy-induced taste alteration scale**

| Item |  |  | Score |  |  |
| --- | --- | --- | --- | --- | --- |
|  | **1** | **2** | **3** | **4** | **5** |
| Factor 1: Decline in basic taste  Have difficulty tasting bitterness  Have difficulty tasting sourness  Have difficulty tasting saltiness  Have difficulty tasting sweetness  Have difficulty tasting umami | 〇 | 〇 | 〇 | 〇 | 〇 |
| Factor 2: General taste alterations  Food does not taste as it should  Everything tastes bad  Have difficulty tasting food  Unable to perceive the smell or flavor of food | 〇 | 〇 | 〇 | 〇 | 〇 |
| Factor 3: Phantogeusia and parageusia  Have a bitter taste in the mouth  Everything tastes bitter  Have a bad taste in the mouth | 〇 | 〇 | 〇 | 〇 | 〇 |
| Factor 4: Discomfort  Have difficulty eating meat  Have difficulty eating oily food  Have a reduced appetite  Have difficulty eating hot food  Bothered by the smell of food  Feel nauseated or queasy | 〇 | 〇 | 〇 | 〇 | 〇 |

Each item scored using a Likert scale of 1–5 where 1 = no difficulty or absence of the disturbance and 5 = maximum difficulty or disturbance.
